# Social disconnectedness and depressive symptoms across age groups: findings from a non-probability sample of employed U.S. adults

**DOI:** 10.3389/fpubh.2025.1716553

**Published:** 2025-11-14

**Authors:** Moka Yoo-Jeong, Lesley E. Steinman, Annie L. Nguyen, Malinee Neelamegam, Ashley L. Merianos, Ali Boolani, Marcia G. Ory, Idorenyin Udoh, Matthew Lee Smith

**Affiliations:** 1 School of Nursing, Bouvé College of Health Sciences, Northeastern University, Boston, MA, United States; 2 Health Promotion Research Center, School of Public Health, University of Washington, Seattle, WA, United States; 3 Herbert Wertheim School of Public Health and Human Longevity Science, University of California, San Diego, San Diego, CA, United States; 4 Department of Population & Community Health, College of Public Health, University of North Texas Health Science Center, Fort Worth, TX, United States; 5 School of Human Services, University of Cincinnati, Cincinnati, OH, United States; 6 Human Performance and Nutrition Research Institute, Oklahoma State University, Stillwater, OK, United States; 7 Center for Community Health and Aging, Department of Environmental and Occupational Health, School of Public Health, Texas A&M University, College Station, TX, United States; 8 Center for Community Health and Aging, Department of Health Behavior, School of Public Health, Texas A&M University, College Station, TX, United States

**Keywords:** social disconnectedness, loneliness, depressive symptom, young adults, midlife adults

## Abstract

**Background:**

Rates of social disconnectedness and depression have intensified in recent years. Yet, little is known about how they relate to one another across different age groups. This study assessed the relationship between social disconnectedness and depressive symptoms among U. S. adults of varying ages using an internet-delivered survey data collected between November 2021 and January 2022 from a non-probabilistic national sample of 2,496 employed adults aged 18–89 years.

**Methods:**

Participants completed Upstream Social Interaction Risk Scale (U-SIRS-13) and the Patient Health Questionnaire short version (PHQ-2). Within each of five age groups (18–29, 30–39, 40–49, 50–59, 60+), descriptive statistics and Pearson’s *r* correlations were calculated for U-SIRS-13 and PHQ-2. Subsequently, logistic regression models were fitted to assess the relationship between the U-SIRS-13 and PHQ-2 (a score of 3 or greater indicated possible depression), controlling for sociodemographic covariates.

**Results:**

The prevalence of possible depression among participants was 31.6%, which ranged from 46.8% (ages 18–29) to 10.5% (ages 60+). U-SIRS-13 and PHQ-2 had significant associations in all age groups (Pearson’s *r* range: 0.283–0.275, *p* < 0.001). Holding sociodemographic covariates constant, higher U-SIRS-13 scores were consistently associated with increased odds of possible depression across age groups (Odds Ratio range: 1.24–1.50, *p* < 0.001). While possible depression was more prevalent among younger age groups (18-29 and 30-39), the relationship between social disconnectedness and possible depression was stronger among older age groups (40–49, 50–59, and 60+).

**Conclusion:**

This finding supports that regardless of age, individuals who experience higher levels of social disconnectedness are more likely to have possible depression Coordinated efforts are needed to address depressive symptomology and facilitate meaningful interactions with others in all age groups.

## Introduction

Recent years have seen a notable increase in the prevalence of depression, social isolation, and loneliness in various age groups in the United States (U.S.) (1, 2). Depression is a pervasive mental health condition that affects millions of people globally across all ages, contributing significantly to the burden of disease (3). The onset of major depression occurs in early or late life. Nearly 40% of individuals experience their first episode of depression before the age of 20, with prevalence peaking during the second and third decades of life (3). Over half of major depression cases occur in older age (age 60 or older), also known as the late onset depression (4). Depression affects well-being and physical health and also increases risky health behaviors including alcohol and substance use (5). Concurrently, current national reports from 2022 estimate that about 25% of U.S. older adults and about 40% of U.S. young adults experience social isolation or loneliness (6). Prior studies also indicate that nearly half of U.S. adults report feeling lonely (7, 8), with most recent 2023 survey showing that nearly 30% of U.S. adults ages 18 and 34 report feeling lonely at least weekly compared to about 17% older adults report loneliness (9). Conversely, older adults ages 65 and older report more social isolation than their younger counterparts (10, 11).

A large body of research has demonstrated that social constructs (e.g., such as social isolation, loneliness, and low social support) are robustly associated with depressive symptoms across age groups (12–14). *Social isolation* typically refers to the objective lack or infrequency of social contacts, such as living alone or having limited interaction with others, while *loneliness* refers to a subjective perception of inadequate social connection (15). Both of these constructs captures important but distinct dimensions of social experience, and together, they have informed much of what we know about the social determinants of mental health. However, these narrower constructs are often used in silo, either on the objective absence of social ties (e.g., social isolation) or the subjective experience of unmet social needs (e.g., loneliness), limiting the understanding of other relevant dimensions of risk that stem from the broader, everyday social interactions that combine multidimensional constructs. More recently, researchers have begun to explore broader, integrative concept of *social disconnectedness*—an umbrella term that encompasses the concepts of social isolation and loneliness as well as social interactions and engagement (16–18)—which has emerged as a critical determinant of physical and mental health (19). Social disconnectedness is a broader construct that encompasses dimensions of both social isolation and loneliness, capturing a wide range of structural and perceived deficits in social engagement and support. Use of this umbrella term may offer greater utility in identifying at-risk individuals, particularly among groups where social risk manifests differently, which is especially relevant for prevention and intervention efforts.

The interplay between social disconnectedness and depressive symptoms is complex and may be bidirectional (12). Existing evidence about the association between social disconnectedness and depressive symptoms suggests that people who are socially disconnected (i.e., feel lonely or isolated, report low social support, and experience strain in their relationships) are more likely to develop symptoms of depression (14, 20). However, depressive symptoms themselves can contribute to reduced social motivation, avoidance of interaction, and negative social cognitions, which in turn may increase the likelihood of further social withdrawal and subsequent disconnection (21, 22). This reciprocal pattern may create a reinforcing cycle in which poor mental health and increased social disconnectedness exacerbate one another over time.

Factors such as the replacement of personal communication channels with technology-mediated ones, the advent of technology communication, changes in family dynamics and structure, and the impact of the COVID-19 pandemic have been implicated in these rising trends (23, 24). Despite the evidence demonstrating a significant relationship between social disconnectedness and depressive symptoms in specific age groups (i.e., adolescents or older adults), there is a paucity of research examining how this association varies across early adulthood to older age (18–89 years), and much of this work has focused on single, narrowly defined constructs. Less is known about how broader construct of social disconnectedness, which may holistically capture a wider array of social interactions, relate to depressive symptoms across age cohorts.

Closing the research gap regarding the age-specific dynamics of social disconnectedness and depressive symptoms may contribute to the broader understanding of social and mental health across the age cohorts. As such, this study seeks to address that gap by examining how the strength of the association between social disconnectedness and depressive symptoms varies across five age groups spanning early adulthood to older age (i.e., 18–29, 30–39, 40–49, 50–59, 60+). Based on prior literature that utilized narrower constructs, we hypothesized that, while the prevalence rates of social disconnectedness and depressive symptoms would vary by age, their association would be significant across all age groups even after controlling for sociodemographic covariates.

## Methods

Data were analyzed from a cross-sectional sample of employed adults collected using an internet-delivered questionnaire. Participants were recruited through Qualtrics Online Panels (25) using a non-probability sampling approach between November 22, 2021, and January 4, 2022. The overall purpose of the study was to identify the wellness of working Americans following the COVID-19 pandemic and identify perceptions about work culture. Given the study focus, eligibility criteria required all participants to be age 18 years or older, a full-time employee, and a resident of the United States. After eligible participants were identified by Qualtrics, they were presented with a link to the online questionnaire, which required acknowledgment of an Institutional Review Board (IRB)-approved information sheet. Participation in the study was voluntary, and participants could choose to stop taking the survey at any time. While attention-check questions were not incorporated into the instrument, initial data quality checks were performed by Qualtrics to ensure participants met eligibility criteria, completed all questionnaire items, and thoughtfully completed the instrument (e.g., time taken to complete, pattern responding, duplicate respondents) (26). Qualtrics maintains a network of managed panels composed of U.S. adults who voluntarily opt in to participate in online surveys. Eligibility for this study required participants to be currently employed, reside in the United States, and be between the ages of 18 and 60. Quotas were applied to approximate national demographic distributions (e.g., age, gender, race/ethnicity, and U.S. Census quartiles). Potential participants were recruited by Qualtrics to complete the online questionnaire. A total of 2,932 participants initiated the questionnaire, of which 2,508 completed the questionnaire (85.6%). Of those, 12 participants were omitted from analyses for missing data on specific variables of interest, resulting in an analytic sample of 2,496 employed adults. The Texas A&M University IRB reviewed and approved all components of this study (#IRB2021-1127 M).

### Measures

#### Depressive symptoms

The Patient Health Questionnaire-2 (PHQ-2) was used to identify depressive symptoms among participants (27, 28). This two-item scale contains the first two items of the PHQ-9, a validated screening measure for depression (29), which measures the two cardinal symptoms of depression: depressed mood and anhedonia. The PHQ-2 asked participants to report the frequency they “felt down, sad, or hopeless” and “had little interest or pleasure in doing things” in the past 2 weeks. Response choices were on a 4-point Likert scale and ranged from “not at all” (scored 0) to “nearly every day” (scored 3). These items were summed, with a total score ranging from 0 to 6. The scores were dichotomized using the recommended cutoff of ≥3, indicating those with possible depression (30). The PHQ-2 was selected for its efficiency and validated use in large-scale population studies, including online and survey-based research, where minimizing participant burden is important. The PHQ-2 has demonstrated strong correlation with the PHQ-9 and high diagnostic accuracy in identifying major depressive disorder across diverse populations, including adults in community and primary care settings (27, 31).

#### Social disconnectedness

The Upstream Social Interaction Risk Scale (U-SIRS-13) was used to identify the risk of social disconnectedness among participants (32, 33). This 13-item scale was developed to specifically assess the broader concept of social disconnectedness among older adults in clinical and community settings (32, 33). asked participants to report the frequency of feeling disconnected in terms of physical opportunities to interact with others and the emotional fulfillment of such interactions (or lack thereof). Response choices were on a 3-point Likert scale, including “never” (scored 1), “sometimes” (scored 2), and “often” (scored 3). Based on the practical scoring recommendations provided by Smith and Barrett (33), each item was then dichotomized based on the directionality of the wording to create items scored as “no risk” (scored 0) and “risk” (scored 1). Items were then summed to generate a continuous score from 0 to 13, with higher scores indicating higher risk for social disconnectedness. Cronbach’s alpha for the U-SIRS-13 in the sample was 0.78, which aligns with reliability coefficients identified in other studies (33). Unlike scales that focus either on loneliness or social isolation, the U-SIRS-13 includes items that assess structural, functional, and quality aspects of social connection (33)—such as frequency of social and religious participation, access to social support, and feelings of companionship or belonging. This multidimensional scale allows for a more comprehensive assessment of individuals at risk for poor social connectedness across multiple life contexts.

#### Sociodemographic covariates

Analyses were performed across five participant age groups (i.e., ages 18–29 years, 30–39 years, 40–49 years, 50–59 years, 60 + years). Age was also analyzed continuously within each age group, respectively. Other sociodemographic characteristics included in analyses were sex (i.e., male, female), ethnicity (i.e., non-Hispanic, Hispanic), and race (i.e., White, Black, Asian, Other/Multiple Races).

### Data analysis

All analyses were performed using the IBM SPSS version 29. Descriptive statistics were calculated for all variables of interest, which were initially compared by participant age group and PHQ-2 score ≥3. When comparing across age groups, chi-square tests were used for categorical variables and one-way ANOVA were used for continuous variables. When comparing across PHQ-2 score ≥3, chi-square tests were used for categorical variables and two-tailed independent sample *t*-tests were used for continuous variables. Cronbach’s alpha coefficients were calculated to identify the reliability of the U-SIRS-13 for the total sample and each age group. Point-biserial correlation coefficients were calculated to identify the strength and direction of relationships between U-SIRS-13 (continuous) and PHQ-2 score ≥3 (dichotomous) for the total sample and each age group. Then, a series of logistic regression models were fitted to assess the associations of U-SIRS-13 and covariates (i.e., age, sex, ethnicity, and race) on PHQ-2 score ≥3 for all participants, then separately within each age group. For each model, PHQ-2 score <3 served as the referent category with statistical significance set at *p* < 0.05. Collinearity statistics were calculated (i.e., Variance Inflation Factor (VIF) and tolerance), which indicated no multicollinearity among independent variables. To account for multiple analyses and reduce risks for Type I errors, a Benjamini Hochberg False Detection Rate of 95% was used (34). All significant findings met these criteria.

## Results

Table 1 reports sample characteristics stratified by age group and possible depression (PHQ-2 score ≥3). Ages ranged from 18 to 89 years. On average (±SD), participants were aged 43.30 (±14.21) years, with 16.9% being ages 18–29 years, 31.2% ages 30–39 years, 16.2% ages 40–49 years, 19.0% ages 50–59 years, and 16.9% ages 60 years and older. About half of the sample (50.3%) was female, and 81.1% identified as non-Hispanic. About 77.4% of participants identified as White, 14.1% were Black, 3.4% were Asian, and 5.2% another race or multiple races. A total of 31.6% participants had possible depression. On average, participants reported a U-SIRS-13 score of 6.92 (±3.40) on a scale from 0 to 13.

**Table 1:** Sample characteristics by age group and possible depression.

Variable	All ages (*n* = 2,496)	Age groups							Possible depression (PHQ-2 = 3+)			
		18–29 Years (*n* = 421)	30–39 Years (*n* = 779)	40–49 Years (*n* = 404)	50–59 Years (*n* = 473)	60 + Years (*n* = 419)	*χ*^2^ or f	*p*	No (*n* = 1708)	Yes (*n* = 788)	*χ*^2^ or *t*	*p*
Age	43.30 (±14.21)	24.91 (±3.11)	33.85 (±2.91)	44.08 (±2.88)	55.31 (±2.68)	65.04 (±4.44)			46.36 (±14.29)	36.66 (±11.55)	18.06	***
Sex							89.16	***			7.37	**
Male	49.7%	43.0%	56.9%	39.4%	40.4%	63.5%			47.8%	53.7%		
Female	50.3%	57.0%	43.1%	60.6%	59.6%	36.5%			52.2%	46.3%		
Ethnicity							16.29	**			14.80	***
Non-Hispanic	81.1%	74.1%	82.3%	83.2%	82.7%	82.1%			83.1%	76.6%		
Hispanic	18.9%	25.9%	17.7%	16.8%	17.3%	17.9%			16.9%	23.4%		
Race							120.51	***			15.54	***
White	77.4%	61.5%	77.9%	78.5%	79.1%	89.3%			79.4%	72.8%		
Black	14.1%	26.6%	13.1%	10.4%	13.3%	7.6%			12.7%	17.0%		
Asian	3.4%	2.9%	4.4%	4.2%	3.8%	1.0%			3.4%	3.4%		
Other Races	5.2%	9.0%	4.6%	6.9%	3.8%	2.1%			4.4%	6.7%		
U-SIRS-13 (range: 0 to 13)	6.92 (±3.40)	7.74 (±2.93)	7.43 (±3.24)	7.01 (±3.35)	6.48 (±3.51)	5.58 (±3.59)	30.17	***	6.09 (±3.48)	8.72 (±2.38)	−21.95	***
Possible depression							241.77	***				
No	68.4%	53.2%	55.1%	71.5%	82.7%	89.5%						
Yes	31.6%	46.8%	44.9%	28.5%	17.3%	10.5%						

*p < 0.05, **p < 0.01, ***p < 0.001; PHQ, Patient Health Questionnaire; U-SIRS, Upstream Social Interaction Risk Scale.

When comparing sample characteristics by age groups, significantly smaller proportions of participants ages 30–39 years and 60 years and older were female. A significantly larger proportion of participants ages 18–29 years identified as Hispanic. In terms of race, a significantly larger proportion of participants ages 18–29 years identified as Black or another or multiple races, whereas a larger proportion of participants ages 60 years and older identified as White. Significantly larger proportions of participants ages 18–29 years and 30–39 years had possible depression. On average, participants of younger age groups reported significantly higher U-SIRS-13 scores.

When comparing sample characteristics by possible depression, participants reporting possible depression were significantly younger than those without depression. Significantly larger proportions of men and participants who identified as Hispanic had possible depression. Relative to those without possible depression, a significantly larger proportion of participants who reported being Black or another or multiple races reported possible depression. On average, participants reporting possible depression had significantly higher U-SIRS-13 scores than those without possible depression.

Table 2 reports the Cronbach’s alpha reliability coefficients for the U-SIRS-13 as well as the point-biserial r coefficients between the U-SIRS-13 and PHQ-2, for all participants and within each age group. For the total sample, the Cronbach’s alpha coefficient for the U-SIRS-13 data was 0.78, and the point-biserial *r* revealed a significantly positive association between the U-SIRS-13 and PHQ-2 (r_pb_ = 0.36, *p* < 0.001). The Cronbach’s alpha coefficients for the U-SIRS-13 data increased across older age groups. The correlation between the U-SIRS-13 and PHQ-2 remained significantly positive across age groups and increased across older age groups.

**Table 2:** U-SIRS-13 internal reliability and correlations between social disconnectedness and possible depression.

Variable	All Ages (*n* = 2,496)	Ages 18–29 (*n* = 421)	Ages 30–39 (*n* = 779)	Ages 40–49 (*n* = 404)	Ages 50–59 (*n* = 473)	Ages 60 + (*n* = 419)
U-SIRS-13 Cronbach’s Alpha	0.78	0.70	0.76	0.78	0.80	0.83
Correlation: Point-Biserial r_pb_ (*p*-value) [U-SIRS-13 & PHQ-2]	0.36 (***)	0.28 (***)	0.31 (***)	0.35 (***)	0.34 (***)	0.38 (***)

*p < 0.05, **p < 0.01, ***p < 0.001; PHQ, Patient Health Questionnaire; U-SIRS, Upstream Social Interaction Risk Scale.

Table 3 reports the binary logistic regression models examining the factors associated with possible depression (PHQ-2 score ≥3) by age groups, controlling for age (continuous), sex, ethnicity, and race. In the model with all participants, each additional unit increase of the U-SIRS-13 increased the odds of possible depression. In this model, each additional year of age decreased the odds of possible depression. Relative to male participants, female participants were less likely to have possible depression. Relative to non-Hispanic participants, Hispanic participants were more likely to have possible depression. Across regression models for each age group, each additional unit increase of the U-SIRS-13 increased the odds of possible depression, with odds ratios increasing across older age groups. For participants ages 18–29 years, Hispanic participants were more likely to have possible depression compared to their non-Hispanic counterparts. In the model for participants ages 30–39 years, each additional year of age decreased the odds of possible depression. In the models for participants ages 30–39 years and 40–49 years, female participants were less likely to have possible depression compared to male participants.

**Table 3:** Factors associated with possible depression by age group.

Variable	All Ages (*n* = 2,496)			18–29 Years (*n* = 421)			30–39 Years (*n* = 779)			40–49 Years (*n =* 404)			50–59 Years (*n* = 473)			60 + Years (*n* = 419)		
	95% CI			95% CI			95% CI			95% CI			95% CI			95% CI		
	OR	Lower	Upper	OR	Lower	Upper	OR	Lower	Upper	OR	Lower	Upper	OR	Lower	Upper	OR	Lower	Upper
U-SIRS-13	1.29***	1.25	1.34	1.23***	1.14	1.33	1.25***	1.19	1.32	1.33***	1.22	1.45	1.37***	1.25	1.50	1.51***	1.33	1.72
Age	0.95***	0.94	0.96	0.95	0.89	1.02	0.92**	0.87	0.97	0.97	0.90	1.06	0.95	0.86	1.04	0.92	0.83	1.03
Male	1.00	–	–	1.00	–	–	1.00	–	–	1.00	–	–	1.00	–	–	1.00	–	–
Female	0.69***	0.57	0.84	1.14	0.75	1.72	0.54***	0.39	0.75	0.52**	0.32	0.84	0.94	0.55	1.59	1.20	0.59	2.45
Non-Hispanic	1.00	–	–	1.00	–	–	1.00	–	–	1.00	–	–	1.00	–	–	1.00	–	–
Hispanic	1.30*	1.02	1.65	1.8*1	1.10	2.99	1.36	0.90	2.04	0.93	0.49	1.77	1.30	0.67	2.51	1.05	0.44	2.49
White	1.00	–	–	1.00	–	–	1.00	–	–	1.00	–	–	1.00	–	–	1.00	–	–
Black	1.12	0.85	1.46	1.14	0.71	1.83	1.26	0.80	1.99	1.01	0.44	2.34	1.70	0.83	3.48	0.93	0.25	3.49
Asian	0.85	0.51	1.42	0.57	0.16	2.01	1.36	0.65	2.84	1.08	0.34	3.46	0.21	0.03	1.70	–	–	–
Other Races	0.99	0.65	1.50	0.68	0.32	1.48	0.92	0.43	1.96	1.59	0.66	3.82	1.72	0.54	5.46	–	–	–-
Nagelkerke R Square	0.284			0.137			0.179			0.213			0.224			0.312		

Referent Group: PHQ<3, *p < 0.05, **p < 0.01, ***p < 0.001; PHQ, Patient Health Questionnaire; U-SIRS, Upstream Social Interaction Risk Scale.

## Discussion

This study assessed the risk prevalence for social disconnectedness and possible depression, and the association between them, across adults in different age ranges. Results provide robust evidence of a positive relationship between social disconnectedness and possible depression across all age groups. Notably, while younger adults (ages 18–39 years) exhibited the highest prevalence of depressive symptoms, the strength of the association between social disconnectedness and depression appeared stronger among midlife and older adults. These findings underscore the importance of age-sensitive strategies to mitigate social disconnectedness as part of mental health promotion efforts.

Aligned with prior studies (35–37), we found that the prevalence of social disconnectedness and possible depression were significantly higher among young adults aged 18–39 years. Their respective prevalence rates gradually decreased across age groups, reaching their lowest points among individuals aged 60 years and older. This finding may suggest the presence of age-related disparities in mental health and social well-being, with younger adults having a higher risk for both social disconnectedness and possible depression. A recent report demonstrates that the health and wellbeing of younger people is largely impacted by the pervasiveness of digital communication and smart devices on daily lives (38). For example, while technology has provided opportunities for consistent communication and connection with others, its ubiquitous nature may generate the need for ongoing stimulation and the feelings of incompleteness without its presence (39). The higher rates of social disconnectedness and depression observed among younger adults in the current study could be attributed to their higher level of technology use (40, 41); however, future studies are needed to examine how technology utilization influences these outcomes across age ranges.

The association between social disconnectedness and possible depression was significant across all age groups, confirming our study hypothesis. This finding supports that regardless of age, individuals who experience higher levels of social disconnectedness are more likely to have possible depression (12, 42, 43). Interestingly, the strength of the positive correlation coefficients between these two factors gradually increased with increasing age (40–49, 50–59, and 60+) and was notably stronger in older age groups. Several factors may contribute to this pattern. First, older adults often experience significant life changes— such as reduced social networks due to loss of loved ones, bereavement, retirement (44, 45), health challenges, and functional limitations (46)—that can disrupt social roles and diminish opportunities for meaningful engagement. As a result, disconnectedness may be felt more acutely and have a stronger emotional impact in later life. The Socioemotional Selectivity Theory posits that as one grows older, they view the time left in life as more limited and, as such, selectively choose to narrow and focus their social relationships that bring meaning and purpose instead of broadening their social ties (47). It might be that as one grows older, the need for meaningful relationships increases, and when this expectation is not reached (i.e., more socially disconnected), individuals may be more likely to experience mental distress (i.e., depressive symptoms). However, given the cross-sectional nature of this study with a convenience sample, future research is needed to complement these exploratory findings and validate these age-related patterns observed in the current study. Second, coping strategies may differ by age group; younger adults may be more likely to use technology to maintain connection, while older adults may rely more heavily on in-person interactions that are disrupted by health or mobility constraints (48). Generational norms around mental health expression and help-seeking could influence how depression is experienced and reported. Older adults, particularly those from historically stoic or self-reliant cohorts, may underreport emotional distress or delay seeking support, potentially amplifying the psychological burden of social disconnectedness (43, 49). These factors highlight the need for age-sensitive strategies that measure and mitigate the impacts of social disconnectedness on mental health across the lifespan.

The association between social disconnectedness and depression across all age groups underscores the need for intervention strategies targeting to reduce social disconnectedness tailored for specific age groups in different life context to improve effectiveness and accessibility. Prior literature suggests a range of risk factors for loneliness and isolation that may differ in magnitude at specific age groups (36, 37). For example, interventions for young adults may focus on skills to enhance relationship-building with friends and coworkers in educational and workplace settings as frequent contact with friends and relationship in workplace mattered more for loneliness in this age group compared to older age groups (50). For midlife adults, interventions could be integrated into workplace wellness or parenting support programs as this age group is faced with changing social roles and work-life-balance (44). As such, practical solutions for these age groups may include integrating mental health resources into employee wellness programs, creating informal peer-support networks, or embedding social connection components into digital mental health apps and employee onboarding processes. For older adults, approaches such as intergenerational activities and digital literacy support may help reduce barriers to connection (51, 52). These approaches could include community-based technology training, telehealth support, or programs that match older adults with digital mentors to reduce barriers to social participation using technology and mitigate the negative consequences of disconnectedness. Interventions that integrate social prescribing, structured group activities, or neighborhood-level social infrastructure (e.g., senior centers, transportation services) may also be effective. Healthcare providers should consider assessing social connectedness as part of routine depression screenings and offering interventions that foster social interaction alongside traditional treatments for depression. Similarly, employers should offer opportunities for more social interaction and approaches to enhance mental health in the workplace. At the policy level, efforts to address digital equity, invest in community-based mental health and aging services, and incentivize intergenerational or workplace-based connection models are warranted. These age-tailored approaches acknowledge that social disconnectedness is not experienced uniformly and that the pathways to prevention and support must align with life stage, access, and context (53).

Our findings revealed a novel result that male participants were more likely to report possible depression compared to female participants, with this significant association driven by younger age groups (ages 30–49). This result contrasts with prior studies that demonstrate that women are more likely to report depression than men, a phenomenon known as the “female preponderance in depression” (54, 55). Traditionally, men were far less likely to report depression or mental health symptoms due to societal expectations and mental health stigma that may provoke masculinity (56). In fact, our oldest age group that was predominantly comprised of White men, reported the lowest rates of possible depression, which aligns with previous research showing that men tend to underreport depressive symptoms (54). As such, the discrepancy in our findings may reflect a generational shift, with younger men becoming more expressive about their mental health as societal stigma surrounding mental health issues for males have decreased with an increase in awareness of its importance (43, 49). Further studies are needed to explore these gendered and age-varying patterns on mental health outcomes across different generations in a greater detail.

Consistent with previous studies (57, 58), participants in the current study who identified as racial and ethnic minorities had higher rates of probable depression. These findings likely reflect a complex interplay of social, structural, and health-related factors. One explanation is that race often serves as a proxy for exposure to systemic racism (59), which has been consistently linked to poorer mental health outcomes (60, 61). These stressors may exacerbate the feelings of hopelessness and emotional distress, further compounding the risk of depression. In addition, structural barriers, such as limited availability of culturally competent clinicians, financial constraints, and systemic inequalities in healthcare access, may contribute to the underutilization of mental health services by these minoritized groups (62). Studies indicate that individuals from these communities are less likely to receive accurate diagnoses and adequate treatment for depression, which may result in unmet mental health needs (63, 64). Stigma surrounding mental health, compounded by historical and ongoing medical mistrust, may further limit health-seeking behaviors for these minoritized groups. Black and Hispanic individuals often face dual stigmatization—both for experiencing mental health issues and for seeking treatment—which may serve as barriers to timely intervention and can lead to misdiagnosis or under-diagnosis of depression (62–64). Stigma, coupled with the historical medial distrust, underscores the need for culturally sensitive approaches to mental health care that directly address the unique challenges faced by these populations. Addressing these systemic factors is warranted for reducing the observed disparities in depression risk and improving mental health outcomes in these marginalized communities across all ages.

There are several limitations to consider when interpreting the study’s findings. It is important to note that the cross-sectional study design precludes inferences about causality. Although prior literature suggests a potentially bidirectional relationship between social disconnectedness and depression, our analysis modeled social disconnectedness as the independent variable and depression as the outcome. Therefore, assessment of the association operating in both directions using longitudinal research designs is warranted to examine causal pathways. Future studies should also consider examination of between- and within-person differences that are reflective of the true developmental and life course changes in the cross-sectional relationship found in this study. All data were self-reported, which may have introduced social desirability or recall biases. Generalizability may be limited because the data were drawn from a non-probability sample of employed U.S. adults recruited via Qualtrics Online Panels. As such, the findings may not generalize to unemployed individuals, those with more severe health or functional limitations, or populations with lower digital access or literacy. These populations may be at elevated risk for social disconnectedness or depression, suggesting that our findings may not be fully representative of the broader U.S. adult population. Additionally, the study’s inclusion requirement of employment status may have skewed the sample toward individuals who were more functionally able or socially integrated, which could have suppressed the prevalence or severity of outcomes of interest. The observed pattern of higher depressive symptoms among men, particularly those aged 30–49 years, should be interpreted with caution given the non-probability sampling strategy of employed Americans. As described previously, this sample may reflect generational or contextual differences in depression reporting, but it may also be influenced by selection biases related to workforce participation and digital access. Moreover, individuals with more severe depressive symptoms and/or social disconnectedness may have been less likely to participate, potentially leading to underestimates of its respective prevalence rates and overestimating the association strength between these variables, especially among the oldest age group (i.e., 60 + years). While about 15% of the current sample was age 60 years or older, there were limited number of participants ages 70 years and older (*n* = 61), which constrained our ability to further stratify analyses by finer age subgroups within the older adult population (e.g., 60–69 years, 70–79 years, 80–89 years). Future research should specifically focus on older adults, particularly those beyond traditional working age, to better understand how aging influences the relationship between social disconnectedness and mental health in later life. Such studies could offer valuable insights into how life stage, retirement, and aging-related shifts in social roles may interact with these variables. Depression was assessed using the PHQ-2, a brief screening tool, which limited the ability to fully capture diagnostic criteria, symptom chronicity, or severity. Although the PHQ-2 is a validated and widely used screening tool, its brevity may underestimate depression prevalence, particularly among older adults or individuals with milder or somatic symptom presentations. Future studies should employ more comprehensive diagnostic tools and inclusive sampling strategies to examine these relationships across broader and more diverse populations. Moreover, race, ethnicity, sex, and age were assessed as discrete categories rather than its intersectional factors in relation to risk for depression (65). Further research assessing the interplay between these various identities on mental health outcomes is needed to gauge more nuanced understanding of the associations to reduce health inequalities (66). The current study used practical scoring recommendations for the U-SIRS-13 (33), which yielded robust findings. However, using alternative U-SIRS-13 scoring methods to explore its relationship with depression may further validate the robustness of observed relationships in the current study. The U-SIRS-13 was originally validated with older adults and has been used with other adult populations (e.g., middle-aged caregivers) (33), thus its use with younger populations in the current study is exploratory and provides preliminary indications of its appropriateness for those ages 18 to 60 + years. Lastly, although we adjusted for several sociodemographic variables, we did not include important confounders such as life stressors, substance use, and personality traits, all of which are known to affect depressive symptoms (67). The omission of these factors may bias the observed associations and limit the internal validity of the findings. Future studies should aim to include a more comprehensive set of covariates to better isolate the unique impact of social disconnectedness on depression.

Despite these limitations, this study’s findings show that there is a significant relationship between social disconnectedness and possible depression across different age ranges, highlighting the importance of multidimensional, upstream social risk factors for mental health. These results underscore the importance of fostering social connectedness and managing depression. Efforts are warranted to understand age-informed intervention strategies to address social disconnectedness and depressive symptoms across the adult life span.

## Data Availability

The raw data supporting the conclusions of this article will be made available by the authors, without undue reservation.
